# Pado, a fluorescent protein with proton channel activity can optically monitor membrane potential, intracellular pH, and map gap junctions

**DOI:** 10.1038/srep23865

**Published:** 2016-04-04

**Authors:** Bok Eum Kang, Bradley J. Baker

**Affiliations:** 1 Center for Functional Connectomics, Korea Institute of Science and Technology, Seoul 136-791, Republic of Korea

## Abstract

An *in silico* search strategy was developed to identify potential voltage-sensing domains (VSD) for the development of genetically encoded voltage indicators (GEVIs). Using a conserved charge distribution in the S2 α-helix, a single *in silico* search yielded most voltage-sensing proteins including voltage-gated potassium channels, voltage-gated calcium channels, voltage-gated sodium channels, voltage-gated proton channels, and voltage-sensing phosphatases from organisms ranging from mammals to bacteria and plants. A GEVI utilizing the VSD from a voltage-gated proton channel identified from that search was able to optically report changes in membrane potential. In addition this sensor was capable of manipulating the internal pH while simultaneously reporting that change optically since it maintains the voltage-gated proton channel activity of the VSD. Biophysical characterization of this GEVI, Pado, demonstrated that the voltage-dependent signal was distinct from the pH-dependent signal and was dependent on the movement of the S4 α-helix. Further investigation into the mechanism of the voltage-dependent optical signal revealed that inhibiting the dimerization of the fluorescent protein greatly reduced the optical signal. Dimerization of the FP thereby enabled the movement of the S4 α-helix to mediate a fluorescent response.

In this age of optically imaging neuronal activity, current versions of genetically encoded voltage indicators (GEVI) offer the potential of monitoring inhibition and activation in a single neuron^1^,^2^,^3^,^4^,^5^,^6^,^7^. However, the ultimate goal is to optically map the activity of multicellular, neuronal circuits involving the fluorescent imaging of tens to potentially thousands of cells. Since the nervous system uses voltage in many different ways to convey and process information, the membrane potential of neuronal cells can vary from hyperpolarizations during neuronal inhibition to depolarizations from synaptic activity and the firing of action potentials. These differing states of membrane potential during the imaging of neuronal circuits complicate the optical signals from fluorescent voltage sensors since it is likely that integrated light signals will come from several cells potentially experiencing different neuronal activities. Restricting the optical response of a GEVI to one type of activity (*i.e*., inhibition) would greatly facilitate the optical analyses of neuronal activity.

Restricting the voltage sensitivity of GEVIs requires a better understanding of the mechanism coupling fluorescent changes to alterations in membrane potential. One of the best GEVIs developed to date is ArcLight which can give an optical signal of nearly 40% ΔF/F upon 100 mV depolarization in HEK cells^2^, nearly 20% ΔF/F for action potentials in dissociated hippocampal neurons^8^, and over 2% ΔF/F when imaging the odor invoked signals in the olfactory bulb *in vivo*^9^. ArcLight consists of four primary domains: a cytoplasmic N-terminus, four transmembrane segments that constitutes the voltage-sensing domain (VSD), a cytoplasmic linker region that fuses a fluorescent protein (FP) to the VSD, and the FP, the optical reporter which resides in the cytoplasm. Mutations to any of these domains can affect the signal size, speed, and voltage sensitivity of the optical signal of the GEVI^2^,^7^,^8^,^10^,^11^,^12^,^13^,^14^. These modifications to ArcLight related probes are an advantage over voltage-sensitive fluorescent organic dyes since in theory the GEVI could potentially be ‘tuned’ to report specific types of neuronal activity by restricting the optical response to specific voltage ranges. For instance, the voltage-dependent fluorescent response of the GEVI, Bongwoori, has a V_1/2_ (the voltage at which the fluorescence change is half of the maximum) near 0 mV that improves the optical resolution of action potentials from sub-threshold depolarizations^8^.

Nature has developed a vast array of voltage-responsive proteins. To improve our ability to optimize the voltage sensitivity, kinetics, and signal size of GEVIs, an *in silico* search strategy was developed to identify potential voltage-sensing proteins since new genomes are routinely being sequenced. The use of a conserved amino acid motif in the second transmembrane segment of the voltage-sensing phosphatase (VSP) gene family enabled the identification of distantly related voltage-sensing proteins including voltage-gated calcium channels (Cav), voltage-gated sodium channels (Nav), voltage-gated potassium channels (Kv), and voltage-gated proton channels (Hv). One VSD identified from this strategy was an uncharacterized Hv from Chinese Liver Fluke, *Clonorchis sinensis*. Fusing this VSD to a pH-sensitive FP, super ecliptic pHlourin resulted in a GEVI named Pado (Korean for wave) that gave a voltage-dependent fluorescent signal and a distinct, pH-dependent fluorescent signal. Biophysical characterization of Pado demonstrated that the voltage-dependent fluorescence change was due to the movement of S4. The pH-dependent signal was due to the Hv channel activity of the VSD.

The voltage-gated proton current provides an easy way to manipulate the internal pH of the cell. Raising the internal pH of the cell did not seem to affect the voltage-dependent optical signal; however, introduction of a mutation to inhibit the dimerization of the FP dramatically reduced the size of the voltage-dependent optical signal. The effect of dimerization was confirmed by testing monomeric FP versions in another GEVI that utilizes a mutated voltage sensing domain from the *Ciona intestinalis* VSP. These results indicate that the mechanism of fluorescence change in response to voltage for Pado consists of the movement of S4 altering the interaction/dimerization of the FP.

## Results

### A conserved motif in the S2 transmembrane segment of the VSD can identify novel, voltage-sensing proteins

**Figure 1:**
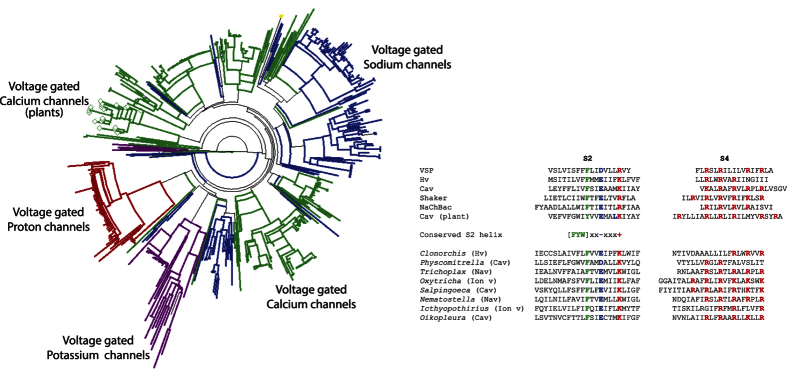
Identification of diverse VSDs. Partial dendrogram of distantly related proteins identified using the VSD from the zebrafish VSP and requiring the sequence [FYW]xx[DE]xxx[RK]. The Hv family of proteins is in red. The Kv family of channels is in purple. The Cav proteins are denoted in green. The Cav nodes ending with a diamond are from plants. Nav channels are in blue. The VSP family and most hypothetical proteins except from plants were removed for demonstration purposes. Comparison of the S2 and S4 transmembrane sequences for the VSP (zebrafish), Hv (human), Cav (mouse), Shaker (Kv – drosophila), NaChBac (Nav - bacteria), Cav plant (*Populus trichocarpa*) are shown as specific examples. The conserved helix sequence was required to be present in a PHI BLAST *in silico* search yielding known and potential novel VSDs from the following organisms and accession numbers: *Ichthyopthirius multifiliis* (freshwater protozoan), EGR29338; *Physcomitrella patens* (moss), XP_001766478.1; *Trichoplax adhaerens* (metazoa), XP_002110559.1; *Oxytricha Trifallax* (ciliated protozoa), EJY83098.1; *Oikopleura dioica* (pelagic tunicate), CBY37723.1; *Salpingoeca rosetta* (choanoflagellate), EGD72607.1; *Clonorchis sinensis* (Chinese liver fluke), GAA49235.1; and *Nematostella vectensis* (starlet sea anemone), XP_001627761.1. The bulky, hydrophobic amino acid in the S2 transmembrane segment is in green. Negatively charged residues in the required bait sequence are in blue, positively charged amino acids are red as are the potential positive charges in the S4 transmembrane segment that respond to voltage.

The S2 transmembrane helix of the VSP gene family contains a highly conserved structural architecture consisting of a well conserved phenylalanine, a negative residue three amino acids downstream followed by a positive residue four amino acids downstream (Fxx[E,D]xxx[R,K], where x is any amino acid)^8^,^15^. This S2 motif is also found in other voltage-sensing proteins^16^,^17^,^18^,^19^,^20^,^21^,^22^. Using the VSD sequence from the Zebrafish VSP protein, a pattern-initiated-hit BLAST search^23^ requiring the presence of the Fxx[E,D]xxx[R,K] motif identified potential VSDs with a diverse distribution of positively charged amino acids in the S4 transmembrane α-helix. Further analyses of these potential VSDs revealed homologies to Hv, Cav, Nav, and Kv as well as two putative mechanosensitive ion channels. Loosening the stringency at the phenylalanine position to be tyrosine or tryptophan ([F,Y,W]xx[E,D]xxx[R,K]) broadened the range of potential VSDs to plants. For demonstration purposes the labels of the nodes in the circular cladogram in Fig. 1 have been removed. Expanded views of the voltage-sensing proteins identified via this search strategy are shown in supplemental Figure 1 demonstrating the range of organisms from mammals to bacteria, algae, and plants. Dendrograms were created using the program, Dendroscope 3^24^. All proteins identified by this search can be found in the datasets 1 and 2 in the supplemental materials.

### A GEVI using the VSD from the Liver Fluke Hv is capable of optically reporting changes in membrane potential

**Figure 2:**
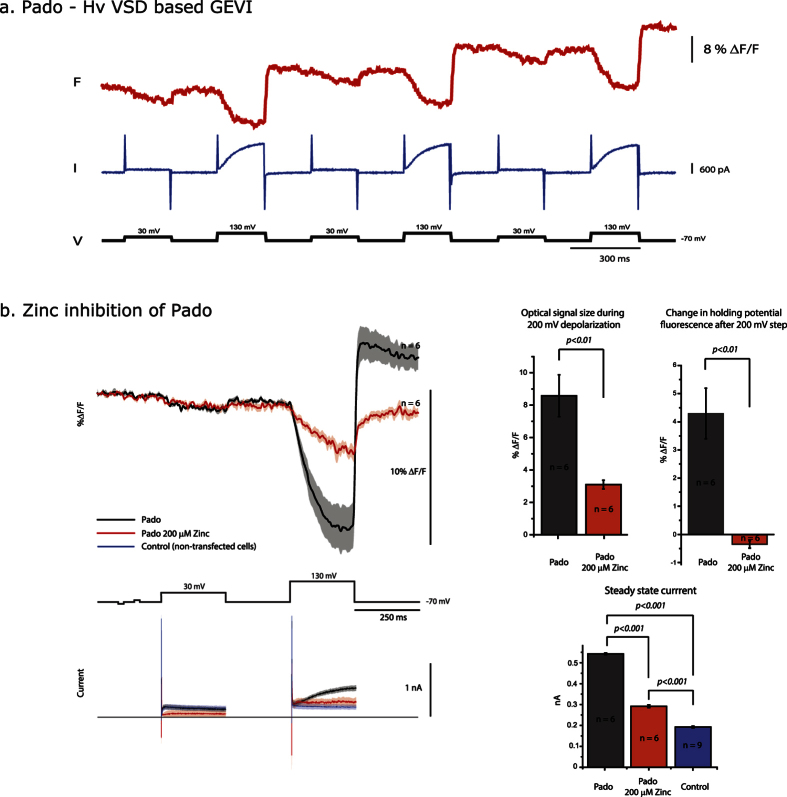
Pado exhibits a voltage dependent optical signal and a voltage dependent current. (**a**) Representative fluorescent (red) and current (blue) traces of an HEK cell expressing Pado in the whole cell patch clamp configuration. The cell was subjected to the voltage command pulses in black. A Gaussian 50 offline filter was used on the fluorescent trace. This trace is from a single trial. The voltage-dependent current seen at the 200 mV depolarization step results in the increase in fluorescence of the cell at the holding potential. (**b**) Pado can be inhibited by Zn^2+^. The darker fluorescence traces represent the average of the optical responses of Pado in the presence or absence of extra-cellular Zn^2+^. The lighter colored traces represent the standard error of the mean (n = 6 for both conditions). Bar graphs represent the fluorescence change from the start of the 200 mV depolarization step to the maximum change recorded. The fluorescence change at the holding potential represents the difference in fluorescent intensities before and after the 200 mV depolarization step. The blue trace depicts the average current from 9 non-transfected HEK 293 cells.

To further validate the S2 search strategy, eight uncharacterized voltage-sensing proteins from different non-mammalian species with a diverse charge distribution in S4 were selected for GEVI testing. These novel GEVIs were created by replacing the transmembrane domains of a previously reported GEVI, CC1^8^ with the corresponding sequences of these new proteins shown in Fig. 1. CC1 is a *Ciona* VSP-based GEVI that trafficks well to the plasma membrane. Since these uncharacterized VSDs are from non-mammalian species, the N-terminus and linker region connecting the FP to the S4 transmembrane segment of CC1 were used to try to maximize the plasma membrane expression. Despite this effort, all constructs exhibited significant intracellular fluorescence. However, one construct based on the putative Hv from the Chinese Liver Fluke (*Clonorchis sinesis*) trafficked well enough to the plasma membrane to demonstrate a voltage-dependent optical signal (Fig. 2). The protein sequence of the novel GEVI is shown in supplemental Figure 2.

This new GEVI yields a modest optical signal, <5% ΔF/F, upon a 100 mV depolarization that increases to nearly 10% ΔF/F upon a 200 mV depolarization step (Fig. 2a) in a single trial. However, analysis of the optical signal during the 200 mV depolarization is confounded by a corresponding jump in the fluorescent baseline. The change in baseline fluorescence is always accompanied by a voltage-dependent current not present during the 100 mV depolarization step. This GEVI, named Pado, contains a pH-sensitive fluorescent protein, Super Ecliptic pHlorin A227D (SE227D)^1^,^25^,^26^ linked to the VSD of a putative Hv channel^22^. If this voltage-dependent current is due to the flow of protons exiting the cell, then the jump in the baseline fluorescence could be due to a change in the intracellular pH. In contrast, a mutation to the VSD in the S1 transmembrane segment of the *Ciona* VSP during the development of the GEVI, Bongwoori, introduced a voltage dependent current that did not affect the baseline fluorescence^8^. The optical response from Pado and the currents therefore suggest that the 100 mV depolarization yields only a voltage-dependent fluorescent signal while the 200 mV depolarization step yields both a voltage-dependent signal and a slower, potential pH-dependent signal.

### Inhibiting the movement of the Hv S4 transmembrane domain reduces the optical signal

To test the Hv channel activity of Pado, HEK 293 cells expressing Pado were voltage-clamped in the presence of extra-cellular Zn^2+^. Zinc is known to inhibit Hv channel activity by binding to the external S3-S4 loop which shifts the gating of the Hv to more positive potentials by inhibiting the movement of S4^22^,^27^,^28^. In the presence of 200 μM Zn^2+^, the optical signal was reduced from an average of 8% ΔF/F to 3% ΔF/F for a 200 mV depolarization, and the voltage-dependent current was also reduced (Fig. 2b). In the absence of the voltage-dependent current, there was no change in the baseline fluorescence. Extracellular Zn^2+^ reduced the movement of S4 such that the channel does not achieve the open state even during a 200 mV depolarization.

In the absence of extracellular Zn^2+^ there is no channel activity at the 100 mV depolarization step, yet there still is an optical signal correlating to the voltage pulse. The 200 mV depolarization step moves S4 to the active state resulting in a voltage-dependent current which also correlates to a jump in the fluorescence baseline. Movement of S4 during the 100 mV depolarization does not reach the open state so that there is no current and the baseline fluorescence remains unchanged. The 200 mV depolarization causes a larger displacement of S4 resulting in the open state of the channel. The optical signal size at 200 mV is larger than the 100 mV step which fits with the movement of S4 increasing to reach the activated state of the channel. As protons then exit causing a rise in the intracellular pH, the fluorescence of SE227D becomes brighter thereby shifting the baseline fluorescence upon the return of the plasma membrane to the holding potential. Inhibition by Zn^2+^ is also reversible. To ensure that the change in the fluorescent signal was due to Zn^2+^ inhibition, a washout experiment was done (Supplemental Figure 3). These effects on the optical signal and the current suggested that the VSD has Hv channel activity that can be inhibited by Zn^2+^. The voltage-dependent optical signal, as well as the voltage-dependent current, can be inhibited by reducing the movement of S4 consistent with Pado exhibiting Hv activity.

### The current-associated optical signal is distinct from the voltage-dependent optical signal

**Figure 3:**
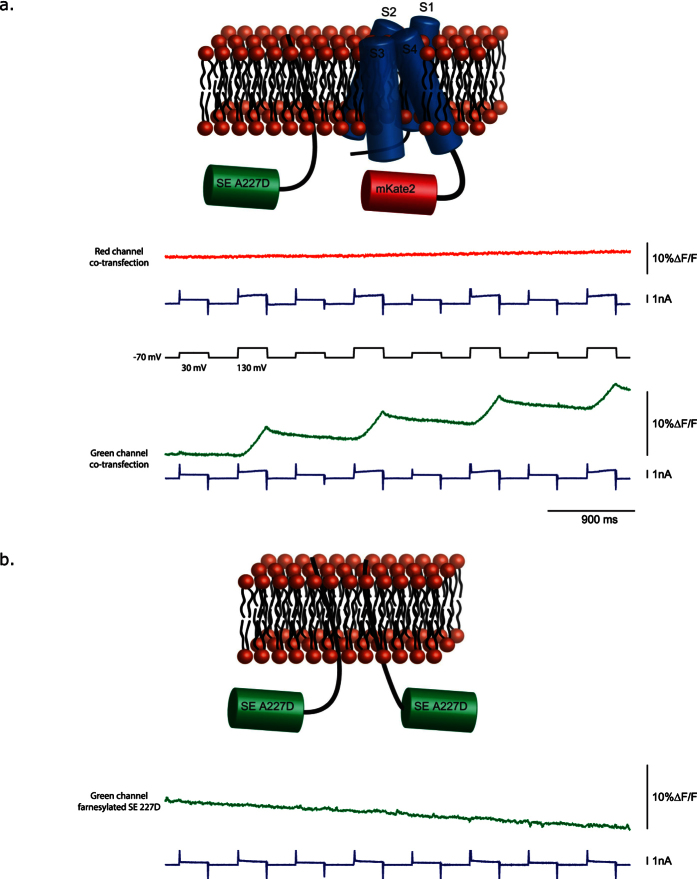
The voltage-dependent optical signal is dependent on the movement of S4. (**a**) The schematic representation of an HEK 293 cell co-transfected with the farnesylated version of SE227D and Pado red, and a representative comparison of the red and green fluorescent signals from the same HEK cell expressing both Pado red and a farnesylated version of SE227D (co-transfection). The red channel represents a single trial with four repetitions of a 100 or 200 mV depolarization step. The green channel represents a subsequent single trial on the same cell. The pH sensitive SE227D shows a rise in the baseline fluorescence corresponding with the voltage-gated current. There is no voltage-dependent downward signal when SE227D is not attached to S4. (**b**) HEK cells only expressing the farnesylated version of SE227D, and a representative trace of an HEK cell expressing only the farnesylated version of SE227D. All traces were filtered offline with a low pass Gaussian 100 filter.

To demonstrate that the rise in baseline fluorescence was due to the pH-sensitivity of SE227D, a truncated version of Pado lacking an FP was co-transfected into HEK cells with a farnesylated^29^,^30^ version of SE227D. While the farnesylated FP expressed well at the plasma membrane, no voltage-dependent current could be detected from the truncated Pado. Only when a red FP, mKate 2^31^, was used to replace the SE227D (rPado) could a voltage-dependent current and pH change be detected (Fig. 3). This result suggested that the carboxyl-terminus is important for the channel activity of this GEVI. The farnesylated SE227D gave an upward fluorescence change that correlated with activation of the current. There was no downward fluorescence change in the green channel in response to membrane depolarization steps. The downward fluorescence change seen in Fig. 2 therefore requires the fusion of the pH sensitive FP (SE227D) to the S4 segment of the VSD to convert voltage changes to an optical signal. The delay in the optical response to the voltage pulse demonstrates the slower kinetics of the current-associated increase in fluorescence (a change in pH). This is especially true for the recovery from the increase in baseline fluorescence. Thus, the pH-sensitive FP is responsible for the increase in the baseline fluorescence. The current-associated signal can be seen when the pH-sensitive FP is associated with the plasma membrane by a lipid anchor and is therefore not directly affected by the movement of S4. Cells expressing only the farnesylated version of SE227D exhibit no change in fluorescence (Fig. 3).

**Figure 4:**
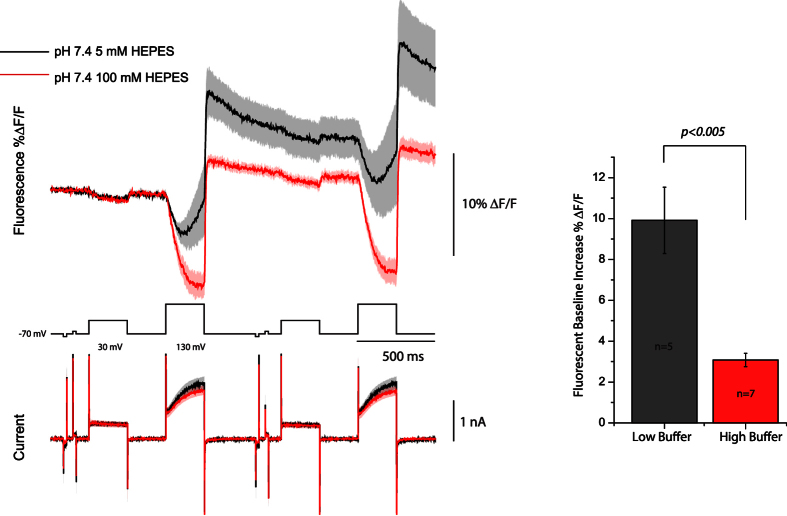
Increasing the buffering capacity of the internal solution reduces the change in the fluorescence baseline. The top traces are an overlay of the optical signal from HEK cells expressing Pado that were subjected to voltage pulses with 100 mM HEPES pH 7.2 internal solution (red) or 5 mM HEPES pH 7.2 internal solution (black). The dark line represents the average from single trial recordings (5 low buffered cells, 7 high buffered cells). Lighter shade is the standard error of the mean. Both fluorescent traces were subjected to an offline, low pass Gaussion 100 filter. Bottom traces are the average current (I). Bar graph represents the change in fluorescence for the holding potential after the 200 mV depolarization activates the current.

Since Pado utilizes the voltage-sensing domain of a putative Hv channel and was inhibited by extracellular Zn^2+^, we hypothesized that the increase in the baseline fluorescence was due to a change in the internal pH. To examine how the pH affects the voltage-dependent optical signal, the buffering capacity of the internal solution in the patch pipette was increased from 5 mM HEPES to 100 mM HEPES. Figure 4 shows that increasing the buffering capacity of the internal solution greatly reduced the increase in the baseline fluorescence after activation of the voltage-dependent current. The consequence of the higher buffering capacity of the internal solution demonstrates that the fast voltage-induced optical change is not due to a change in internal pH but to the movement of S4.

### Pado is capable of sensing the difference in pH across the plasma membrane

**Figure 5:**
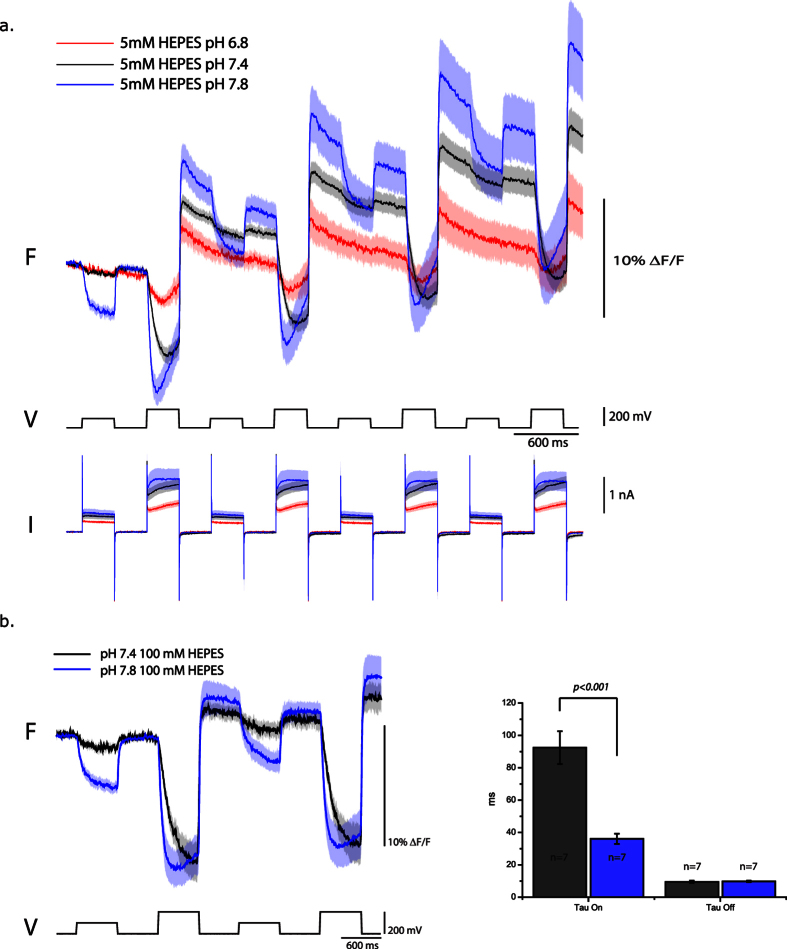
Increasing the intracellular/extracellular pH difference alters the voltage response of Pado. (**a**) Top traces compare the optical response of HEK cells expressing Pado that were voltage clamped with extracellular solution at pH 6.8 (red trace), pH 7.4 (black trace), or pH 7.8 (blue trace). The dark line is the average of the response from 3 cells for each condition. Lighter shade is the standard error of the mean. (**b**) Cells were also tested with higher buffering capacity solutions to show that the speed of the response is also affected by increasing the pH difference across the plasma membrane. Each cell was only tested at one pH condition. The standard error of the mean is in a lighter shade. All cells were voltage clamped at a holding potential of −70 mV with an internal solution of pH 7.2. Bar graph represents the kinetics of the optical signal for the 200 mV depolarization step.

A remarkable feature of Hv channels is the ability to sense the difference between the internal and external pH levels^22^,^28^,^32^. By increasing the difference between the internal and external pH, the voltage-sensitivity of the channel is shifted to more negative potentials. To determine if Pado would exhibit a similar characteristic, HEK cells expressing Pado were voltage clamped and subjected to external bath solutions at pH 7.4 or 7.8. As can be seen in Fig. 5, the optical signal from cells exposed to the higher external pH gave a larger optical signal for both the 100 and 200 mV depolarization steps. Lowering the external bath solution to a pH of 6.8 reduced the fluorescence change of the 200 mV depolarization (Fig. 5). The kinetics of Pado are also affected by the pH difference across the plasma membrane. The τ_on_ is increased nearly 3 fold when the external pH is raised from 7.4 to 7.8, but had little effect on the τ_off_ rates.

### The voltage-dependent optical signal is not affected by the pH-dependent optical signal

**Figure 6:**
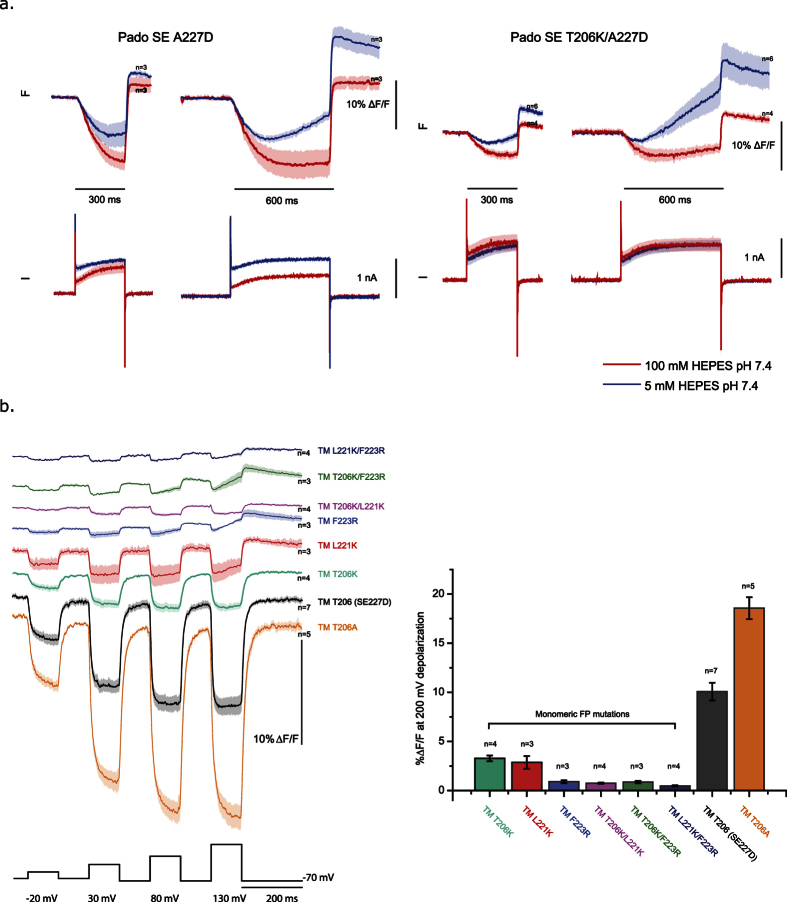
Increasing the internal pH does not inhibit the voltage-dependent optical signal but dimerization of the FP does. (**a**) Comparison of the fluorescent response from HEK 293 cells expressing Pado with varying lengths of 200 mV depolarizations. Blue trace is the average of cells voltage-clamped in 5 mM HEPES internal buffer. Red trace is the average of cells voltage-clamped in 100 mM HEPES internal buffer. Light shade is standard error of the mean. The left traces are from cells expressing Pado. The right traces are from cells expressing Pado with the 206K mutation in SE 227D that inhibits the dimerization of the FP. The T206K mutation in the FP reduces the voltage-dependent signal by ~50%. (**b**) Comparison of *Ciona* VSP-based GEVI (TM) containing mutated versions of SE227D. Three monomeric mutations were introduced into the FP as a single mutation or in combinations. Numbers of the mutation are based on the SE227D amino acid position. Optical traces are color coded for the mutations to the FP. All fluorescent traces were subjected to an offline, low pass Gaussian 100 filter. Bar graph represents the average of the absolute fluorescent signal for a 200 mV depolarization in HEK 293 cells.

The mechanism by which a GEVI with a single, cytoplasmic FP is capable of changing fluorescence in response to changes in membrane potential is not well understood. Since Pado was capable of sensing the pH difference across the plasma membrane, we wondered if the sensor used a similar mechanism to sense changes in membrane potential. Posing this question in a different way, could the pH change inside the cell affect the voltage-dependent fluorescence signal. To test the effect of internal pH on the voltage-dependent signal, the length of the 200 mV depolarization was increased. As the pulse length is increased, the internal pH will continue to rise and eventually overcome the opposing fluorescent signal due to voltage (Fig. 6a, supplemental Figures S4–S6). Independent of the length of the depolarizing step there was a voltage-dependent change in fluorescence. This can be seen by the jump in the optical signal at the end of the voltage pulse regardless of the pulse length. The response to voltage does not appear to be affected by the change in intracellular pH. The movement of S4 is still capable of eliciting an optical signal even during changes in intracellular pH.

### Inhibiting dimerization of the FP reduces the optical signal upon membrane depolarization

How does the movement of S4 mediate a change in the fluorescence of a cytosolic FP? With the intracellular pH having a negligible effect on the voltage-dependent signal, two potential mechanisms exist. Either the FP is interacting with the plasma membrane or with other FPs from neighboring probes. To test the second possibility, a mutation that favors the monomeric version of GFP was introduced into Pado. Figure 6a shows the optical responses of Pado fused to a monomeric T206K variant of SE227D. The voltage-dependent optical signal has been decreased when the dimerization of the FP is inhibited, but both versions of the FP are capable of responding to pH (Fig. 6a and supplemental Figure S7).

The A206K mutation converts GFP from a dimer to a monomer^33^. Super Ecliptic pHlorin also has a mutation at that position (A206T). Whether Super Ecliptic pHlorin is a dimer or monomer is not known, however when the T206K mutation is introduced into Pado the fluorescence response was reduced by nearly two thirds for a 200 mV depolarization from roughly 15% ΔF/F to 5% ΔF/F (Fig. 6a). This result implicates the need for the dimerization/interaction of the FP in order for the movement of S4 to elicit a change in fluorescence.

To ensure that the dimerization of the FP did not just affect an Hv-based GEVI, versions of SE227D with monomeric mutations were fused onto the Triple Mutant GEVI that uses a modified VSD version of the *Ciona* VSP^8^. The VSD from the *Ciona* VSP has the added advantage of not having a voltage-gated current capable of confounding the fluorescent signal. In addition to the A206K, there are two more mutations that convert GFP from a dimer to a monomer^33^. The two other monomeric mutations are L221K and F223R. Again, when the FP contained those individual, monomeric-favoring mutations the optical signal was reduced. A further reduction of the optical response (<1% ΔF/F) was achieved by introducing combinations of the monomeric mutations into the FP. Interestingly, when the T206A mutation is introduced into the FP which reverts that position to the original GFP sequence the optical signal doubles. These results suggest that SE227D has a reduced affinity for dimerization than wild type GFP but can still at least weakly dimerize in the pseudo-2 dimensional space constriction of the plasma membrane since the monomeric mutations are somewhat cumulative in their effect (Fig. 6b).

### Optical mapping of gap junctions and following pH waves with Pado

**Figure 7:**
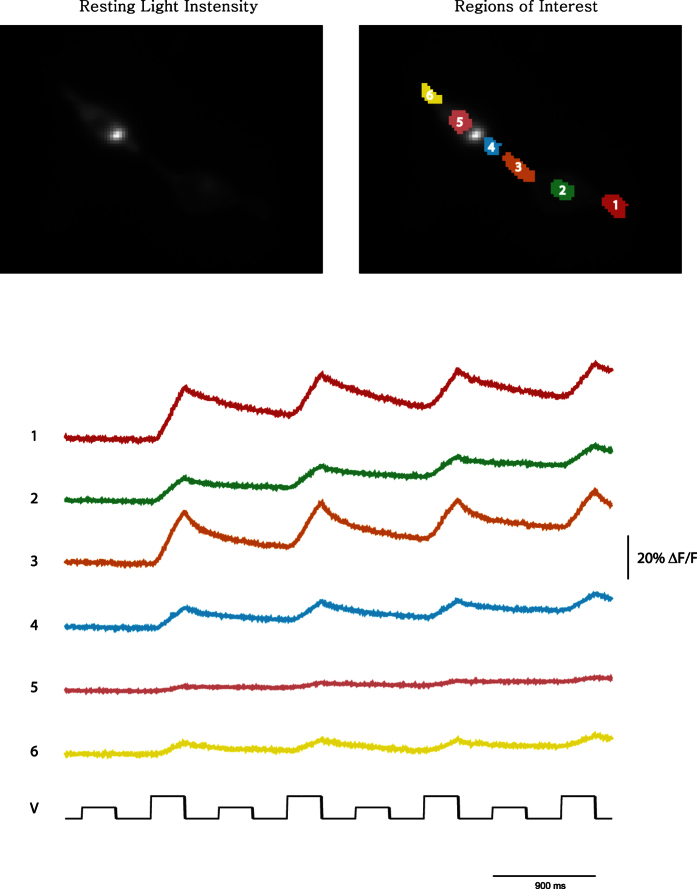
Affecting the fluorescent intensity in a neighboring cell via gap junctions. The upper left panel shows the resting light intensity of two HEK 293 cells expressing Pado (SED227A). The dimmer, lower cell was subjected to whole cell patch clamp and depolarized with 100 and 200 mV pulses. The optical traces below refer to the region of interests depicted in the upper right panel. While the patched cell showed a larger optical signal, all regions showed an increase in fluorescence upon the 200 mV depolarization suggesting that Pado can optically map gap junctions.

HEK cells make gap junctions between neighboring cells. To investigate Pado’s ability to change the pH in neighboring cells via gap junctions, the FP (SE227D) was mutated back to the original 227A which abolished the voltage-dependent signal so that only the pH dependent optical signal would be seen. Figure 7 shows two HEK 293 cells with different expression levels of Pado (SE227A). The dimmer cell was voltage clamped under whole cell configuration and subjected to multiple depolarizations of 100 or 200 mV. Both cells exhibit an increase in fluorescence that corresponds to the activation of the proton current. Since these two cells are electrically coupled, it is possible that the fluorescence change in the neighboring cell is due to activation of the proton channel activity of Pado. However, the clamped cell shows a larger fluorescence change which seems to migrate towards the neighboring cell (compare region of interest #4 to #6 in Fig. 7). Whether the channel activity of the probe in the neighboring cell is activated during the voltage clamp via gap junctions or protons in the neighboring cell are traveling down the concentration gradient through gap junctions, the fluorescence of neighboring cells can be affected via gap junctions. Pado is capable of optically mapping the presence of gap junctions. In addition, Pado is capable of following the pH wave through a cell (supplemental Figures S4–S6). Since some of the expression is internal, Pado can optically report the change in pH throughout the cell. Not surprisingly, cell volume has a dramatic effect on the pH wave.

## Discussion

Remarkably, a conserved charge distribution in relation to a bulky hydrophobic residue spanning two turns of the S2 α-helix enabled the identification of many different types of voltage-sensing proteins from distantly related species in a single search. The F-X-X-(E/D)-X-X-X-(R/K) sequence in the S2 transmembrane domain is highly conserved among most voltage-sensing proteins (phenylalanine can sometimes be tryptophan or tyrosine). The S4 α-helix also contains conserved positive amino acids except that the number and distribution of these residues are more conserved within each type of voltage sensing protein family. For instance, the amino acid sequence in S4 for the Hv family consists primarily of R_1_-X-X-R_2_-X-X-R_3_^34^. The pattern is R_1_-X-X-R_2_-X-X-I_3_-X-X-R_4_-X-X-(R/H)_5_ for S4 of the VSP family^8^,^34^. Nav, Kv, and Cav also have different distributions of positive charges in S4^34^ (Fig. 1). Search strategies that weight the positive charges in S4 therefore bias the results towards one type of voltage-sensing protein family. By forcing the presence of the conserved S2 sequence and allowing for the variation of charges in S4, thousands of uncharacterized VSDs were identified from diverse voltage-sensing gene families from unicellular and multicellular phyla. One of the VSDs tested from this search strategy resulted in a novel GEVI proving that the S2 domain is capable of casting a wide net in the search for new voltage-sensing proteins. While the challenge to traffick these proteins to the plasma membrane remains an issue^35^, a GEVI utilizing the VSD from the Chinese Liver Fluke Hv protein generated a biosensor capable of optically reporting changes in membrane potential as well as changes in the internal pH level. While it is tempting to try to measure absolute pH change, the variability of internal fluorescence and the difficulty of precise pH calibrations present substantial challenges to this endeavor. However, estimates can be made. Using ionophores to change the internal pH from 7.0 to 7.5 more than doubled the fluorescence of the cell (170% ± 40%, N = 20, supplemental Figure 7) which sets an upper limit on the pH change induced by the activation of Pado in this study.

Pado is more than a biosensor. In addition to its optical properties it is also capable of mediating internal pH changes via its Hv activity. Ideally, a GEVI should not affect the physiology of the system to be studied. However, the proton conductivity of Pado has been invaluable for separating and understanding the voltage-dependent fluorescence change from pH induced fluorescent changes. A 100 mV depolarization does not illicit channel activity but yields a modest fluorescent signal. Increasing the depolarization pulse to 200 mV yields a larger optical signal than the 100 mV depolarization and activates the channel. An increase in the optical signal as S4 moves from the sub-activation state (or multiple closed states, see Bezanilla^36^) to the activated state implies that the optical signal is a direct measurement of S4 movement. While other factors cannot be completely ruled out, the more movement of S4 results in a larger voltage-dependent optical signal. Inhibition of the optical signal by Zn^2+^, a characteristic unique to Pado, strengthens this hypothesis since extracellular Zn^2+^ is known to immobilize the movement of S4^27^. Correlation of S4 movement with fluorescence change was documented in the first generation of GEVIs^37^,^38^,^39^,^40^,^41^. Inhibition of the optical signal by preventing the movement of S4 changes this correlated observation into a causative one. A recent study resulting in a new GEVI, ArcLightning, also concluded that the movement of S4 results in the fluorescence change of the probe^14^. Furthermore, the voltage-dependent optical signal proved to be distinct from the pH-dependent optical signal since we were able to decouple them by replacing the pH sensitive FP, SE227D, with mKate 2 or by increasing the buffering capacity of the internal solution. A previous study showed that VSFP3.1-mOrange that contains an FP with a pKa of 6.5 was still able to optically report hyperpolarizations of PC12 cells suggesting that pH was not a factor^4^. Here, we report a rapid increase in fluorescence despite the length of the activating voltage pulse that was always observed upon a return to the holding potential demonstrating that Pado is capable of responding to voltage concomitantly during changes in internal pH (Figs 2 and 6a).

The pH sensitivity of the FP in a GEVI is not sufficient for large optical signals, but may be a requirement. SE227D is one of the best FPs for voltage imaging. Optical signals of 20 to 40% ΔF/F/100 mV depolarization^2^,^8^ suggest that pH sensitivity plays an important role. However, when SE lacks the A227D mutation the signal is only about 1% ΔF/F/100 mV^1^. Other pH sensing FPs such as eYFP also give poor voltage-dependent optical signals. When eYFP is fused to the *Ciona* VSD of ArcLight, the optical signal does not exceed 2% ΔF/F/100 mV depolarization^1^,^13^. Yet, mutagenesis of eGFP to give optical signals similar to SE227D resulted in a pH sensitive version of eGFP^13^. All FPs in GEVIs with a single, cytoplasmic FP that give a large voltage-dependent optical signal are pH sensitive, but not all pH sensitive FPs give large signals.

Pado demonstrates that both pH and voltage can affect the fluorescent intensity of SEA227D but in distinct ways. The pH effect is slow. The voltage effect is fast. The pH effect does not require dimerization of the FP. The voltage effect is enhanced by dimerization of the FP. Since the linker length and composition of the amino acid sequence between the VSD and the FP are able to affect the voltage-dependent fluorescent signal^1^,^2^,^7^,^10^, the orientation of the FP seems to be involved in mediating the optical signal. If orientation of the FP matters, that would also imply an interaction between neighboring FPs of two GEVIs. To test the effect of dimerization of the FP on the voltage-dependent optical signal, a mutation that favors the monomeric FP was introduced into Pado. The A206K mutation in eGFP reduces the affinity of the FP to dimerize^33^. The monomeric version of SE227D reduces the voltage-dependent optical signal likely due to the negative charge of 227D being too far away from the neighboring chromophore. Moving a negative charge along the outside of the barrel may affect the protonation state of the chromophore in a transient way which couples the movement of S4 to changes in fluorescence. Since the spectral characteristics of the monomeric and dimeric versions of eGFP are similar^33^, the alteration of the dimer interface may alter the environment of the chromophore during the movement of S4. Introducing monomeric mutations into a VSP-based GEVI also reduced the optical signal during voltage depolarizations (Fig. 6b). Exploration of this interaction may enable the generation of GEVIs with larger optical signals and assist the development of red-shifted GEVIs since most red-shifted FPs are monomeric^42^,^43^,^44^.

Finally, having specific cellular expression of a pH fluorescent sensor that can mediate changes in intracellular pH offers the possibility to study pH regulation and monitor proton diffusion through gap junctions. Expression of Pado enables the study of proton transport at varying internal pH levels. In addition, activation of Pado’s Hv activity in brain slice could potentially monitor the diffusion of protons through gap junctions^45^. Activating the Hv activity of Pado expressed in a neuron (or glia) will change the internal pH resulting in an increase in the fluorescence of the patched cell. Diffusion of protons from electrically coupled cells should also increase the fluorescence of neighboring cells enabling the fluorescent mapping of electrical synapses. While the voltage required to activate Pado may be too severe for neuronal cells, the ability to shift the Hv activity to more negative potentials by increasing the external pH of the bath solution offers researchers a straightforward way to control the internal pH with an immediate, optical feedback.

## Materials and Methods

### *In silico* search strategy

The amino acid sequence from the first three transmembrane domains (S1–S3) from the *Ciona intestinalis* VSP was used as a bait sequence in a PHI BLAST search^23^ requiring the presence of the following amino acid pattern: [FYW]xx[E,D]xxx[R,K], where x is any amino acid. Transmembrane helix domains were predicted using the TMHMM Server for predicting trans-membrane helix regions (Center for Biological Sequence Analysis, Technical University of Denmark)^46^,^47^ and then aligned using ClustalW in MEGA5.1^48^.

### Plasmid design and construction

Eight novel constructs were developed utilizing the putative S1–S4 transmembrane domains of the following genes: *Ichthyopthirius multifiliis* (freshwater protozoan), EGR29338; *Physcomitrella patens* (moss), XP_001766478.1*;Trichoplax adhaerens* (metazoa), XP_002110559.1; *Oxytricha Trifallax* (ciliated protozoa), EJY83098.1; *Oikopleura dioica* (pelagic tunicate), CBY37723.1; *Salpingoeca rosetta* (choanoflagellate), EGD72607.1*; Clonorchis sinensis* (Chinese liver fluke), GAA49235.1; and *Nematostella vectensis* (starlet sea anemone), XP_001627761.1. The cytosolic N-terminus and the linker region between S4 and the FP were based on the CC1 construct^8^. The DNA sequences were optimized for expression in mammalian cells, flanked with Bam HI and Eco RV restriction sites and synthesized by Integrated DNA Technologies (San Diego, CA). The VSD in the CC1 plasmid was replaced with these novel VSDs using the Bam HI and Eco RV sites. The resulting constructs contained the FP, Super Ecliptic pHluorin A227D (SE227D), first reported in the GEVI, ArcLight^1^. The red fluorescent version of Pado was generated by replacing Super Ecliptic pHluorin with mKate 2^31^,^49^. The farnesylation chain was added onto SE227D through PCR amplification with primers BK06 (5′-atgtgCTCGAGctattaGGAGAGCACACACTTGCAGCTCATGCAGCCGGGGCCACT CTCATCAGGAGGGTTCAGCTTAGATCTGAGTCCGGATTTGTATAGTTCATCCATGCCATGTGTAATCCC-3′) and HP017 which integrated EcoRV and XhoI restriction sites used to insert the PCR amplified product into pcDNA3.1 hygro + (Invitrogen).

The mutations in SE227D were made through directed mutagenesis with one or two step PCR. Mutations close to the carboxyl terminus of the SE227D (L221K and/or F223R) only needed one PCR with sense primer A227Forward (5′-GAACAAAAGAAGATATCGCATGAGTAAAGGAG-3′)and anti-sense primers BK40 (5′-TCTAGACTCGAgtcaTCATTTGTATAGTTCATCCATGCCATGT GTAATCCCATCAGCTGTTACAAACTCcttAAGGACCATGTGGTC-3′) for L22 1K, BK42 (5′-TCTAGACTCGAgtcaTCATTTGTATAGTTCATCCATGCCATG TGTAATCCCATCAGCTGTTACtctCTCcttAAGGACCATGTG-3′) for the L221K and F223R, and BK43 for F223R. The mutations at T206 required 2 step PCR with sense primer A227Forward and reverse primer BK24 (5′-CTAGACTCGAgtcaTCATTTGTATAGTTCATCC-3′) for the two extreme ends and BK22(5′-CTGTTTACAACTTCTAagCTTTCGAAAGATCCC-3′) BK23(5′-GGGATCTTTCGAAAGctTAGAAGTTGTAAACAG-3′) for the T206K mutation, BK25(5′-CTGTTTACAACTTCTgccCTTTCGAAAGATCCC-3′) BK26(5′-GGGATCTTTCGAAAGggcAGAAGTTGTAAACAG-3′) for the T206A mutation, and BK36(5′-CTGTTTACAACTTCTgatCTTTCGAAAGATCCC-3′) BK37(5′-GGGATCTTTCGAAAGatcAGAAGTTGTAAACAG-3′) for the T206D mutation. All constructs were verified by sequencing before use (Cosmogenetech).

### Patch Clamp Fluorometry

HEK293 cells were maintained in DMEM (GIBCO) + 10% fetal bovine serum (GIBCO) and seeded onto #0 coverslips (Ted Pella Inc.) coated with poly-l-lysine (Sigma). Transfections were carried out with lipofectamine 2000 (Invitrogen) using the manufacturer’s instructions. Transfected cells were patched within 24 hours. HEK293 cells on coverslips were kept at 35 °C by a heater and perfused with bath solution composed of 150 mM NaCl, 4 mM KCl, 2 mM CaCl_2_, 1 mM MgCl_2_, and 5 mM D-glucose and buffered with 5 mM HEPES. NaOH was used to adjust the pH. Pipette electrodes were pulled from capillary tubing (1.5/0.84 mm; World Precision Instruments) on a micropipette puller (P-97; Sutter Instruments). The pipette solution contained 120 mM K-aspartate, 4 mM NaCl, 4 mM MgCl_2_, 1 mM CaCl_2_, 10 mM EGTA, 3 mM Na_2_ATP and 5 mM HEPES adjusted to pH 7.2 with KOH.

The high buffer bath solution was 100 mM NaCl, 3 mM KCl, 0.5 mM MgCl_2_, 1 mM CaCl_2_, 3 mM Glucose, 100 mM HEPES. High buffer internal solution contained 75 mM K Aspartate, 3 mM NaCl, 3Mm MgCl_2_, 1 mM CaCl_2_, 5 mM EGTA, 3 mM Na_2_ATP and 100 mM HEPES. The high buffer bath solution was adjusted to desired pH with NaOH while the internal solution was adjusted with KOH. ZnCl_2_ was added to the bath solution without any adjustments to the composition of the solution at a concentration of 200uM. For the zinc inhibition experiments, zinc containing solution was manually placed with a 1 mL pipette into the chamber after manual removal of the regular bath solution. HEK293 cells were recorded in whole-cell patch configuration under voltage clamp at −70 mV resting membrane potential which was controlled by a HEKA EPC10 amplifier (HEKA).

The transfected cells were imaged on an Olympus inverted IX71 microscope (Olympus Korea) with a 60 × 1.35 numerical aperture oil-immersion lens (Olympus). The illumination was provided by a 75 watt Xenon arc lamp (Cairn). The microscope filter cube, optimized for GFP, GFP-30LP-B contained an excitation filter at 472 nm (FF02-472/30-25), a dichroic mirror at 495 nm (FF495-Di03-25 × 36), and a long-pass emission filter at 496 nm (FF01-496/LP-25) (Semrock). The filter cube for mKate 2 imaging contained a band pass excitation filter at 561 nm (FF01-561/14-25), a dichroic at 575 nm (FF01-609/54-25), and long pass emission filter at 578 nm (Di02-R561 -25 × 36) (Semrock). The objective C-mount image was demagnified by an Optem zoom system A45699 (Qioptiq) LINOS projected onto a 80 × 80 e2v CCD39 chip of the NeuroCCD-SM 80 camera which was responsible for our imaging taken at 1kfps (RedShirtImaging). The entire apparatus was mounted on a Vibraplane anti-vibration table (Kinetic Systems, Boston MA. Minus K Technology, Inglewood CA). The illumination arc lamp, amplifier, mechanical shutter, and manipulator were placed on a different platform and did not contact the anti-vibration surface.

### Fluorescence Intensity Measurements

HEK293 cell expressing the desired construct were transfected after seeding on #0 coated coverslips (Ted Pella Inc.) The cells were exposed to gramicidin D (Sigma) at a concentration of 50 μM. The stock gramicidin was prepared by dissolving in DMSO (Sigma) at a 200× concentration (10 mg/ml). The perfusion system was paused while the gramicidin was applied and allowed to perforate the cell membranes at 37 °C for 20 minutes. Fresh bath solution was then perfused into the chamber to wash out the remaining gramicidin. Bath solutions of varying pH were then applied to the chamber and the cells imaged for fluorescence intensity.

### Data analysis

Optical signal recordings were analyzed using Neuroplex software (Redshirt Imaging) and Origin8.6 (Origin Labs). Off-line low-pass filtering is indicated in the figures. All the traces of Pado are from single trials. Other constructs (farnesylated SE227D) tested were internally averaged during the recording for improved signal to noise.

## Additional Information

**How to cite this article**: Kang, B. E. and Baker, B. J. Pado, a fluorescent protein with proton channel activity can optically monitor membrane potential, intracellular pH, and map gap junctions. *Sci. Rep.* **6**, 23865; doi: 10.1038/srep23865 (2016).

## Supplementary Material

Supplementary Figures

Supplementary Figure 5

Supplementary Dataset 1

Supplementary Dataset 2
