# Supplementary material for: Pado, a fluorescent protein with proton channel activity can optically monitor membrane potential, intracellular pH, and map gap junctions
Source: Sci Rep. 2016 Apr 4;6:23865. doi: 10.1038/srep23865 (PMC4878010; doi:10.1038/srep23865)
Supplement: Supplementary Figures [file srep23865-s1.pdf]

Pado, a fluorescent protein with proton channel activity can optically monitor membrane potential, intracellular pH, and map gap junctions.

Bok Eum Kang and Bradley J. Baker

Supplemental figure 1.

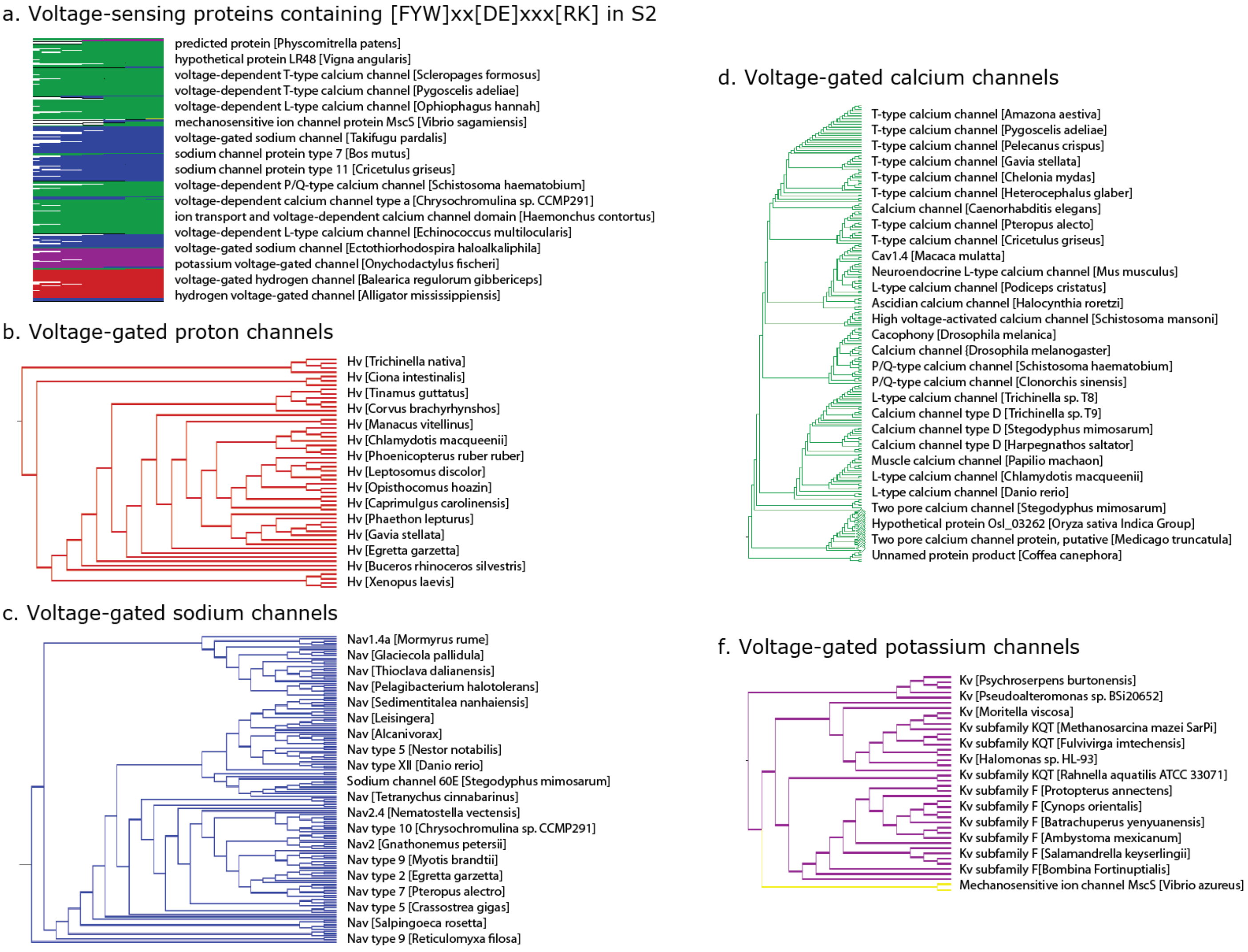

S1. Partial dendrograms of the proteins identified using the VSD of the zebrafish VSP. a. Dendrogram of the more distantly related proteins (below threshold as determined by the default parameters of BLAST which is why the VSP family is not shown). Putative calcium channels are in green, putative sodium channels in blue, putative proton channels in red, putative potassium channels in purple, and two putative mechanosensitive ion channels are in yellow. The dendrogram has been condensed with sparse node labeling for display purposes. Most hypothetical proteins have been removed with the exception of those from plants. **b**. Expansion of the putative proton channel subset from the dendrogram in **a** with sparse

labeling. **c**. Expansion of the putative sodium channel subset from the dendrogram in **a** with sparse labeling. **d**. Expansion of the putative calcium channel subset in **a** with sparse labeling. Nodes denoted with a diamond refer to plant proteins. **f**. Expansion of the putative potassium channels and the two putative mechanosensitive ion channels subsets in **a** with sparse labeling. Color coding is the same as in **a**.

Supplemental figure 2.

```
MEGFDGSDFS PPADLVGVDG AVMRNVVDVT INGDVTAPPK AAPRKSESVK KVHWNDVDQG
PSEKPETRQE ERIDIPEISG LWWGENEHGV DDGRMEIPTT GVGRVQFRIR AVIDHLGMRV
FHLTIVILCC LDGLLVICVL LLEIEALKLK SSSELRPKLV NVQFAIECCS LAIVFLFVVE
IPFKLWIFGC RMFFHNWLEI IDALVCLVSF AADSYSIIHH TLHHYTNTDR PQSHMQTSQS
NATRGLTEHF PVEESAASNT IVDAAALLIL FRLWRVVRIF YSHQQMKASS RRTISQNKRR
YRMSKGEELF TGVVPILVEL DGDVNGHKFS VSGEGEGDAT YGKLTLKFIC TTGKLPVPWP
TLVTTLTYGV QCFSRYPDHM KRHDFFKSAM PEGYVQERTI FFKDDGNYKT RAEVKFEGDT
LVNRIELKGI DFKEDGNILG HKLEYNYNDH QVYIMADKQK NGIKANFKIR HNIEDGGVQL
ADHYQQNTPI GDGPVLLPDN HYLFTTSTLS KDPNEKRDHM VLLEFVTADG ITHGMDELYK
```

S2. Pado protein sequence. The entire amino acid sequence for Pado is represented. The black sequence corresponds to the *Ciona* N-terminal VSP sequence and linker sequence between S4 and the FP. The red sequence represents the Hv VSD from *Clonorchis sinensis*. The green sequence depicts the SE227D fluorescent protein. The underlined amino acids refer to the required motif in S2 or the positive arginines in S4.

Supplemental figure 3.

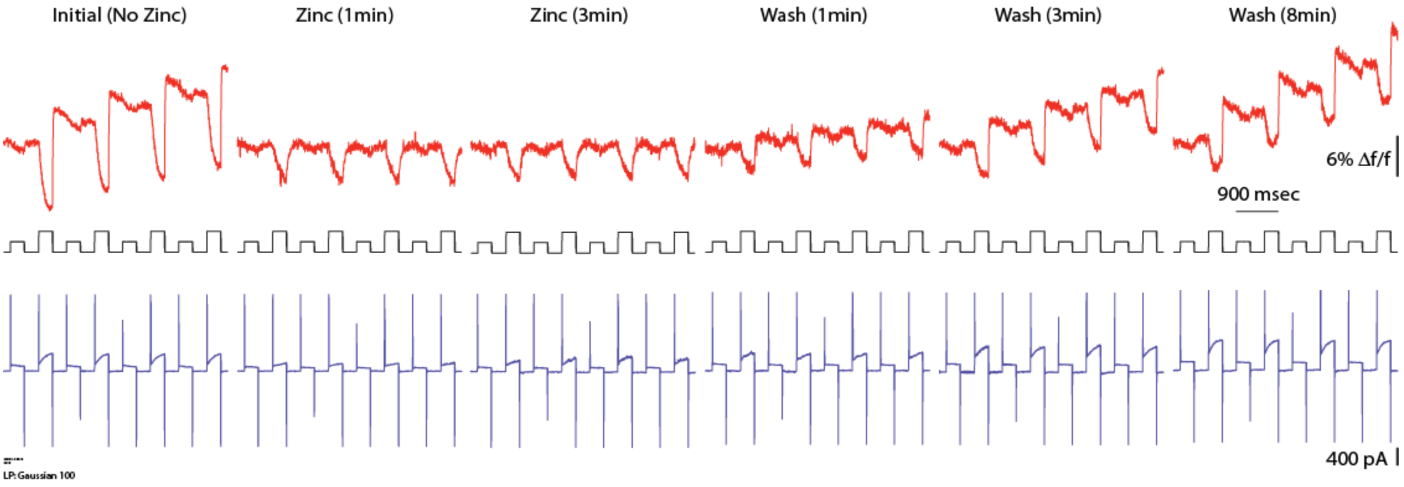

S3. Zinc inhibition is reversible. A representative HEK 293 cell expressing Pado was voltage clamped and subjected to 100 mV and 200 mV depolarization steps (black trace). Fluorescent trace is in red. Current trace is in blue. Upon addition of 200 μM Zn^{2+} the signal size of the fluorescence drops and the voltage-associated current is reduced. Removal of extracellular Zn^{2+} results in the return of the voltage-dependent current and the associated increase in baseline fluorescence. All traces are from the same cell with a low pass Gaussian 100 filter.

Supplemental figure 4.

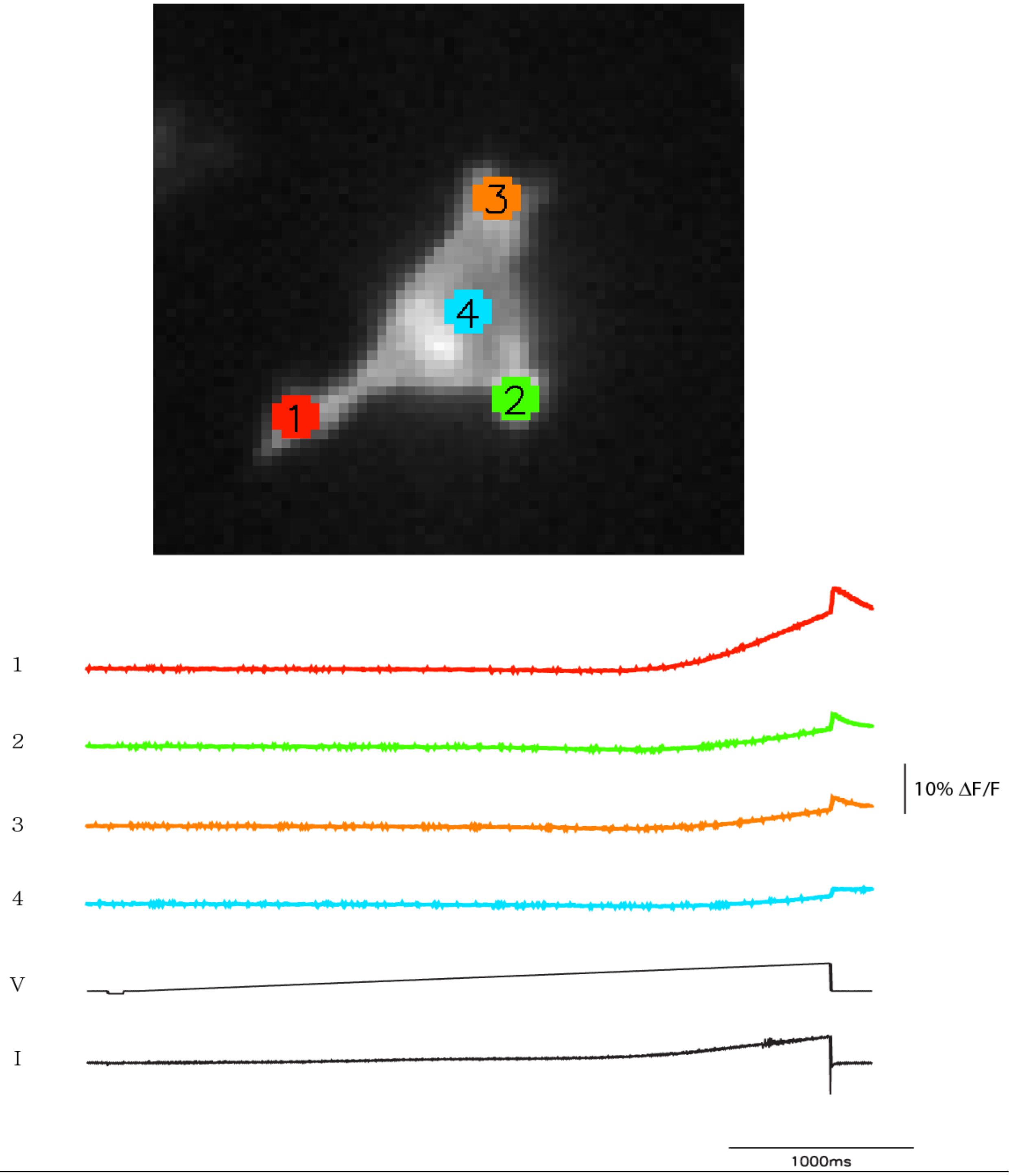

S4. Activating Pado with a voltage ramp protocol. Single trial recording of an HEK expressing Pado. The voltage was ramped from -70 mV to +130 mV. At roughly +80 mV a voltage-gated current can be seen which correlates to an increase in fluorescence that is more pronounced at the edges of the cell.

S5. Movie of HEK cell shown in S1 depicting membrane depolarization followed by intracellular pH increase. Each pixel was normalized to the maximum and minimum fluorescent level during the entire recording and plotted to a pseudo-color range where blue is dimmest and red is brightest. A spatial average of 3x3 pixels was used as was a Gaussian low pass filter of 50 hertz. While the recording was done at one kilohertz, every tenth frame was used to generate the movie. This is a single trial recording.

Supplemental figure 6.

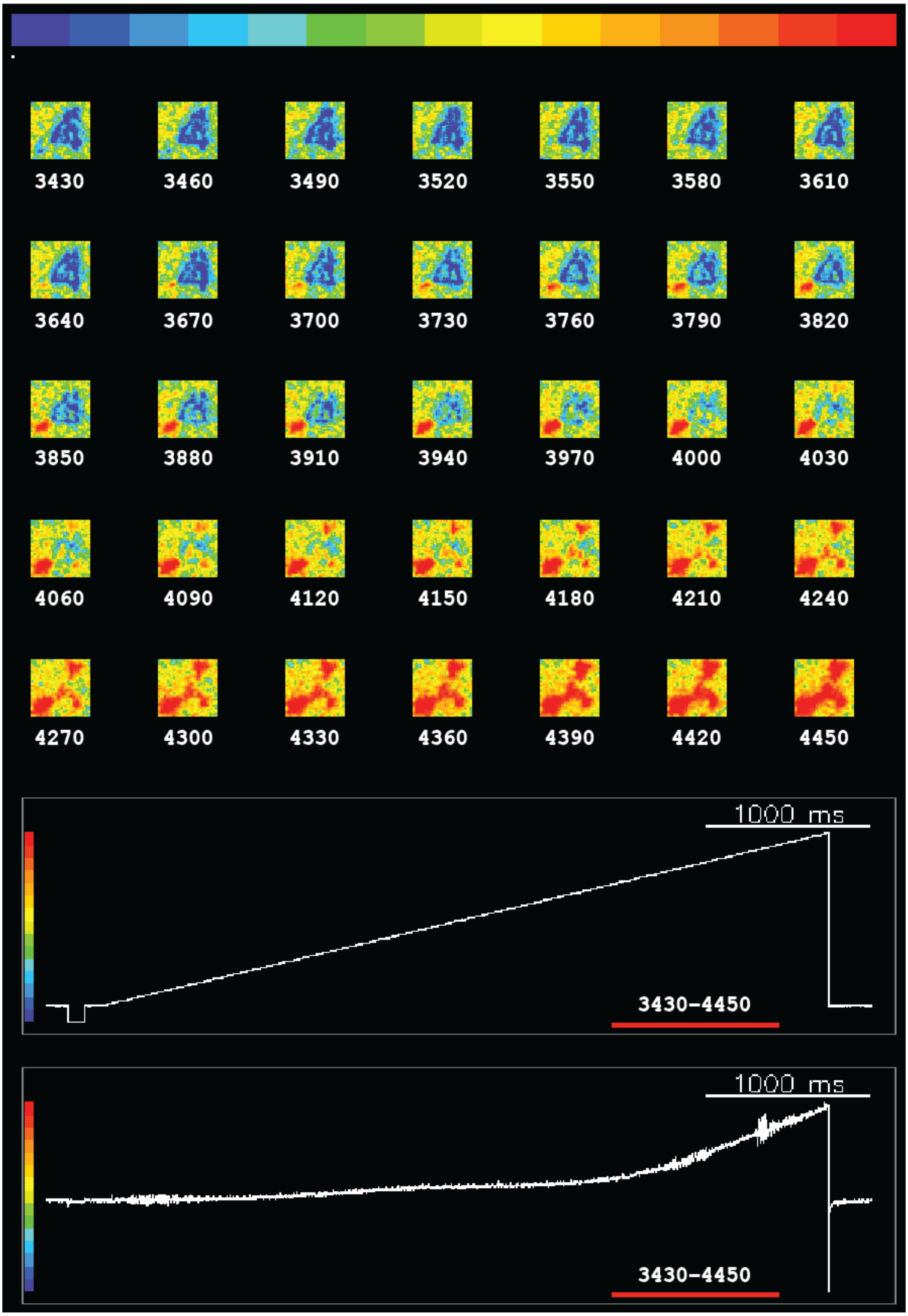

<u>**S6. Optically following the pH wave induced by activation of Pado.**</u> Thirty millisecond intervals are

displayed from frames 3430 to 4450 from the recording in the supplemental figure S4. These frames show the depolarization of the membrane potential as reported by Pado in a reduction of fluorescent intensity followed by the increase in intracellular pH due to the activation of Pado. The pseudo-coloring is as in supplemental figure S5. Below is the voltage clamp protocol with an initial holding potential of -70 mV that was ramped to a final value of 130 mV. Red bar depicts the range of the frames displayed. The current is also shown below demonstrating that the increase in fluorescence correlates with the presence of a voltage-dependent current. Note that the pH changes initially at the three corners of the cell and converges in the middle.

Supplemental figure 7.

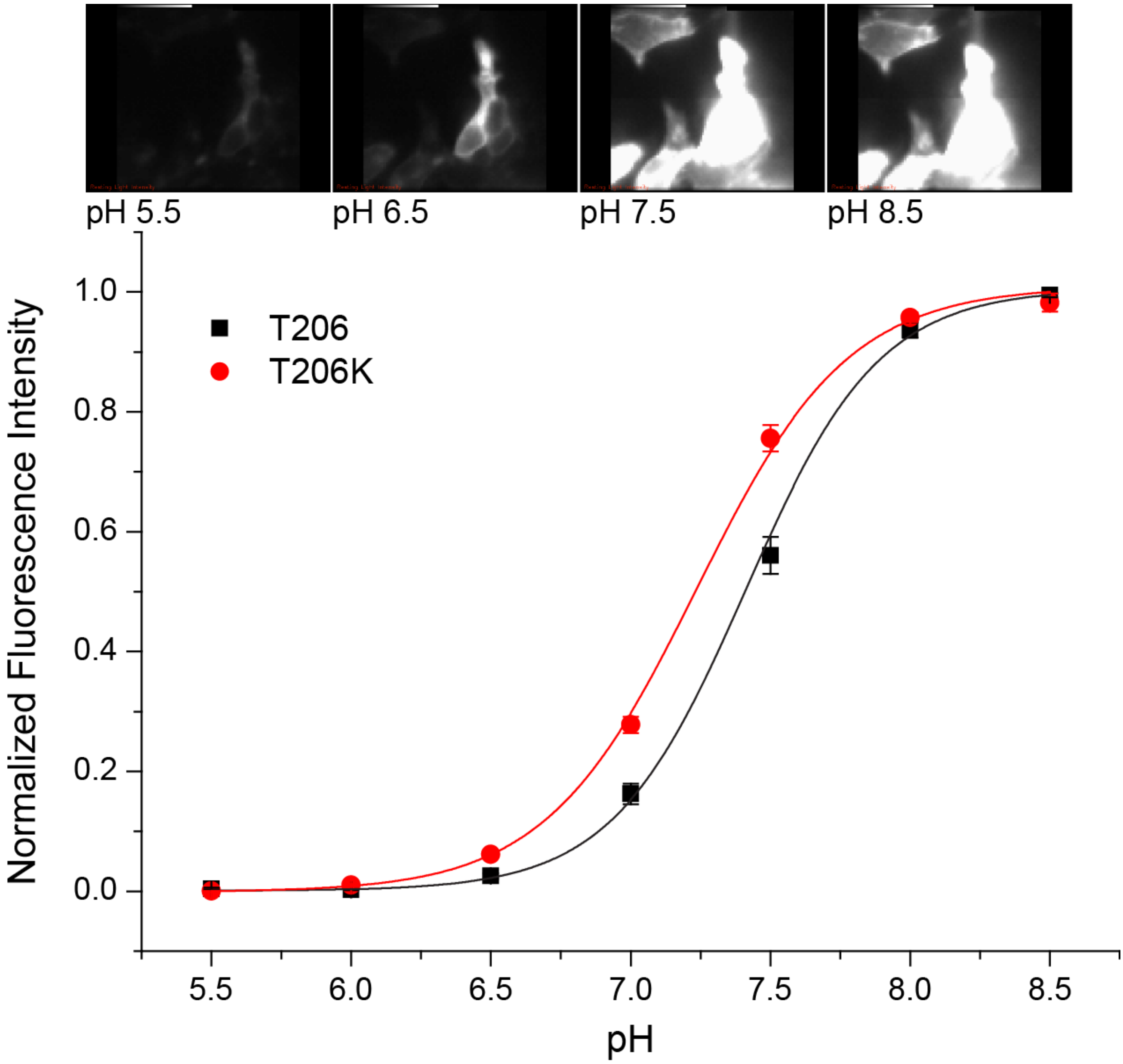

S7. Imaging the fluorescence change of SE227D and SE227D/T206K in response to pH. Twenty cells

expressing the fluorescent protein SE227D or SE227D/T206K were subjected to the ionophore gramicidin D to equilibrate the intracellular solution to the pH of the extracellular bath solution. A slight change in the pKa of T206K was observed from 7.4 to 7.2. Fluorescent images are of T206K.
